# Bridging the Gap: Patient Navigation Increases Colonoscopy Follow-up After Abnormal FIT

**DOI:** 10.14309/ctg.0000000000000307

**Published:** 2021-02-22

**Authors:** Gregory E. Idos, Joseph D. Bonner, Shida Haghighat, Christina Gainey, Stacy Shen, Ashwini Mulgonkar, Karla Joyce Otero, Christine Geronimo, Maria Hurtado, Caitlin Myers, Jennifer Morales-Pichardo, Doron D. Kahana, Paul Giboney, Stanley Dea

**Affiliations:** 1 City of Hope National Medical Center, Duarte, California, USA;; 2 Keck School of Medicine, University of Southern California, Los Angeles, California, USA;; 3 Los Angeles County Department of Health Services, Los Angeles, California, USA;; 4 Charles R. Drew University of Medicine and Science, Los Angeles, CA, USA.

## Abstract

**INTRODUCTION:**

Recent studies indicate low rates of follow-up colonoscopy after abnormal fecal immunochemical testing (FIT) within safety net health systems. A patient navigation (PN) program is an evidence-based strategy that has been shown to improve colonoscopy completion in private and public healthcare settings. The aim of this study was to evaluate the effectiveness of a PN program to encourage follow-up colonoscopy after abnormal FIT within a large safety net hospital system.

**METHODS:**

We established an enterprisewide PN program at 5 tertiary care hospitals within the Los Angeles County Department of Health Services system in 2018. The PN assisted adult patients aged 50–75 years with an abnormal FIT to a follow-up colonoscopy within 6 months. PN activities included initiating referral for and scheduling of colonoscopy, performing reminder phone calls to patient for their upcoming colonoscopy, and following up with patients who did not attend their colonoscopy. We assess the effectiveness of the PN intervention by comparing follow-up colonoscopy rates with a period before the intervention.

**RESULTS:**

There were 2,531 patients with abnormal FIT results (n = 1,214 in 2017 and n = 1,317 in 2018). A majority were women (55% in 2017 vs 52% in 2018) with a mean age of 60 ± 6.2 years. From a previous mean of 163 days without PN in 2017, the mean time from abnormal FIT to colonoscopy with PN improved to 113 days in 2018. The frequency of colonoscopy completion with PN increased from 40.6% (n = 493) in 2017 to 46% (n = 600) in 2018.

**DISCUSSION:**

After the introduction of the PN program, there was a significant increase in patients undergoing follow-up colonoscopy after abnormal FIT and patients were more likely to undergo colonoscopy within the recommended 6 months.

## INTRODUCTION

Colorectal cancer (CRC) is the third most common cancer in the United States and the second leading cause of cancer-related deaths in the United States (1,2). Despite the high burden of disease associated with CRC, significant advances in CRC screening and surveillance have led to improvements in early detection and survival (3). Effective screening surveillance programs not only prevent CRC through identification and resection of precancerous lesions but also aim for early detection of CRC, such that potentially curative treatment can be offered.

Fecal immunochemical testing (FIT) is a widely accepted and implemented first-line screening tool. However, the effectiveness of FIT-based CRC screening is dependent on patients with an abnormal result completing timely diagnostic evaluation (4). At the Los Angeles County Department of Health Services (LAC-DHS), which serves more than 750,000 residents throughout the LA County region, the process for colonoscopy completion includes several steps and requires coordination between primary care providers, gastroenterologists, endoscopy schedulers, and patients. Providers must identify patients with abnormal FIT results and refer patients for diagnostic colonoscopy; the healthcare system must facilitate scheduling; and patients must adhere with surveillance recommendations. Previous data from safety net hospitals in other states suggest that nearly half of patients with abnormal FIT fail to undergo diagnostic colonoscopy (5).

Delays or barriers to colonoscopy after abnormal FIT lead to increased risk of developing CRC (4). Safety net populations that face barriers including lack of insurance coverage, transportation issues, and the need for interpreters are delayed in accessing colonoscopy and timely medical care (6–9). Known barriers specific to CRC screening include confusion about correctly completing FIT and fear of sedation and complications associated with colonoscopy (10). Patient navigation (PN) has emerged as an important intervention to reduce cancer disparities by addressing barriers to cancer care (11–13). General characteristics of PN include the following: (i) assisting patients to identify and overcome barriers; (ii) facilitating patients' access to clinical services; and (iii) for cancer screening, ensuring adherence to screening guidelines, reducing the number of patients lost to follow-up, and improving timeliness of diagnosis and treatment (13).

There is limited published research on the effectiveness of PN for CRC screening. Originally developed to address racial and socioeconomic disparities in breast cancer outcomes (14), PN programs have been shown to improve completion of screening colonoscopy in safety net populations and demonstrated cost-effectiveness (15–17). Within LAC-DHS, a complex and fragmented healthcare system, PN was designed to increase the frequency of follow-up colonoscopy after abnormal FIT by expediting result disclosure and colonoscopy scheduling. Our current study aims to evaluate a PN program designed to improve adherence with a follow-up diagnostic colonoscopy within the recommended 6-month timeframe from test result.

## METHODS

### Study design and patient population

This is a quality improvement study performed within the LAC-DHS healthcare system. LAC-DHS is an integrated safety net healthcare system consisting of 19 community clinic sites, 4 hospitals, and 2 multispecialty ambulatory care centers. LAC-DHS annually cares for 750,000 unique patients using an integrated electronic health record (EHR) and electronic consultation (eConsult) service. The study analysis cohort included men and women between the ages of 50 and 75 years with an abnormal FIT within the first 6 months of 2017 and 2018. Colonoscopy data were collected through July 2019.

### Data sources

LAC-USC is supported by the Online Real-time Centralized Health Information Database (ORCHID) as its primary EHR platform, which is linked to clinical laboratory data and gastroenterology (GI) procedure reporting databases. For all individuals with and abnormal FIT, demographic information, colonoscopy reports, and pathology reports were extracted from ORCHID.

### CRC screening

**Figure 1.**
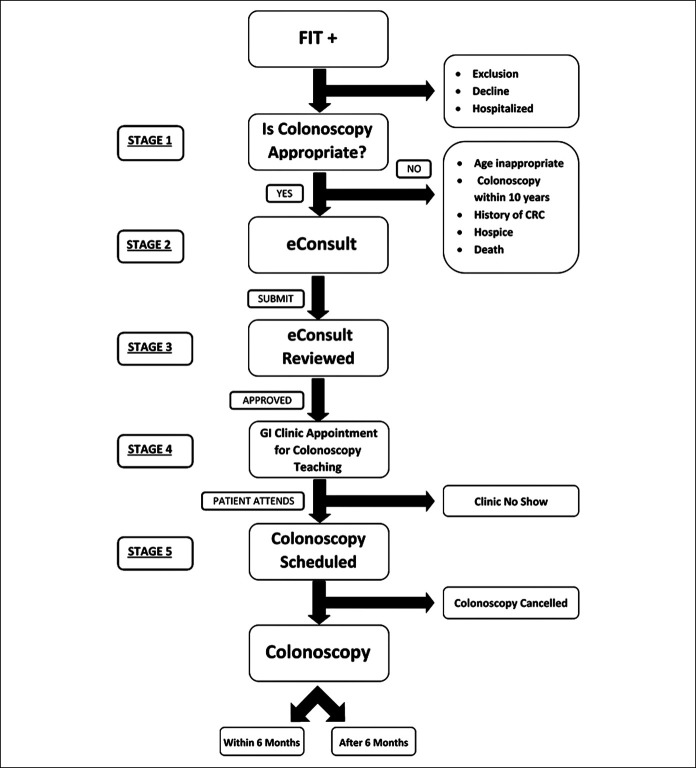
Fecal immunochemical testing (FIT) to colonoscopy flow diagram. GI, gastrointestinal.

Since 2013, FIT was established as the primary CRC screening modality for average-risk patients in the LAC-DHS network. Aligned with United States Preventive Services Task Force guidelines for CRC screening, the DHS defines “average risk” as asymptomatic patients aged 50–75 years without any of the following: (i) personal or family history of CRC or advanced polyp, (ii) underlying hereditary genetic syndrome associated with increased risk of CRC, or (iii) personal history of inflammatory bowel disease. Patients are provided a FIT kit with return postage at a point-of-care visit (Figure 1). The FIT test is performed at home and then mailed to a central laboratory within the LAC-DHS network. The laboratory uses a qualitative FIT platform OC sensor (Eiken Chemical, Tokyo, Japan) with a cutoff of 50 ng HgB/mL of stool. A patient with an abnormal FIT result is flagged and entered in the primary care provider's inbox for review. Colonoscopy is recommended as follow-up for those with an abnormal FIT or for patients at increased risk of CRC (e.g., family history, personal history of polyps, and genetic syndrome).

### From abnormal FIT to colonoscopy

LAC-DHS uses an eConsult platform for GI clinic referrals. A staff gastroenterologist typically evaluates referrals within 48 hours, and based on their recommendation the patient is scheduled for a group colonoscopy class or clinic appointment. The class or clinic appointment, which is offered in multiple languages, explains the meaning of their abnormal FIT and instructs the patient on the risks, benefits, and steps to prepare for the colonoscopy. The procedure is scheduled at the conclusion of the class or clinic appointment. Providers are instructed to refer patients with increased cardiovascular or sedation risk of further evaluation.

### Patient navigation

In this study, PN refers to the practice of navigating a patient from abnormal FIT to colonoscopy. Beginning in 2018, a PN program was designed to increase the rate of CRC screening and expedite patients with an abnormal FIT to colonoscopy by acting an intermediary between primary care and colonoscopy services. The PN program consisted of nurse practitioner or physician navigators within the GI departments that covered 5 LAC-DHS hospital centers and 2 ambulatory care centers. Navigators primarily worked with patients through telephone. Provided with a list of patients with abnormal FIT results on a weekly-to-monthly basis, PNs (i) communicated with the primary care provider to initiate referral for colonoscopy services, (ii) informed and educated patients about the colonoscopy procedure based on their abnormal FIT results, (iii) scheduled patients for precolonoscopy education session, (iv) assisted in scheduling patient colonoscopy within a 6-month timeframe from abnormal FIT, (v) performed reminder call to patients about their upcoming colonoscopy, and (vi) followed up with patients who did not appear for their colonoscopy. The PN is an independent entity who works with the colonoscopy services unit and is outside the referring physician's office.

### Data collection

Data were collected from patients with abnormal FIT using the ORCHID EHR and eConsult system and manually reviewed by 2 gastroenterologists (SD and GI). The primary outcome was completion of diagnostic colonoscopy within 6 months of an abnormal FIT, consistent with LAC-DHS Expected Practice. A diagnostic colonoscopy was considered complete only if the colonoscopy report documented cecal intubation and did not indicate poor colonic preparation. Data collected included the documented date of colonoscopy, referral date of colonoscopy, colonoscopy location and patient travel distance to that location, colonoscopy findings, and pathological findings. Cecal intubation rates are not reported separately for this cohort. However, cecal intubation rates have been reported as >95% across DHS endoscopy sites without significant deviation. Categories for colonoscopy/pathological findings were based on the most advanced finding: normal colonoscopy, at least 1 nonadvanced tubular adenoma, at least 1 advanced tubular adenoma (tubular adenoma greater than or equal to 1 cm, tubular adenoma with tubulovillous features, or tubular adenoma with high-grade dysplasia), adenocarcinoma, or other findings. For patients who did not have a colonoscopy after abnormal FIT, we reviewed and documented whether a colonoscopy request was made and the date of the request.

### Distance calculation

The distance between each patient and their respective colonoscopy facility was calculated using BatchGeo, an online tool for visualizing location data using the Google Maps Geocoding Application Programming. Distances were measured using the home zip code for each patient, as documented in the EHR, and the complete street address of the LAC-DHS designated facility where the colonoscopy was to be performed. To discount the variability of traffic patterns, straight line distance was used to calculate the distance between geographic points.

### Statistical analysis

We used frequencies in proportions or mean values and SDs to describe patient demographic and clinical characteristics. We tested for associations between patients who received a colonoscopy referral for abnormal FIT and the proportion of those patients who underwent a diagnostic colonoscopy within 6 months. We performed Cox regression analysis to compare time from abnormal FIT to diagnostic colonoscopy among patients without PN in 2017 and with PN in 2018. We tested for statistically significant frequency differences in demographic variables between the adherent and nonadherent groups and whether the frequency differences varied by period in which the PN intervention was active. Continuous variables included the differences in average age and the distance from colonoscopy location to patient address.

## RESULTS

### Study population

During the study period, 41,312 FIT tests were performed. A total of 2,531 patients with abnormal FIT results were included in the study, of which there were n = 1,214 in 2017 and n = 1,317 in 2018. A majority of our cohort were women (55% in 2017 and 52% in 2018), and the mean age of those with an abnormal FIT was 60 ± 6.2 years (range 50–75 years of age). Participants were diverse racially/ethnically: 57% were Hispanic, 12% Non-Hispanic white, 14% black, and 10% Asian (Table 1).

### Predictors of colonoscopy

In all, 1,340 (53%) colonoscopies were completed out of 2,531 referrals, n = 682 in 2017 and n = 658 in 2018. Overall, 1,191 (47%) patients did not undergo a colonoscopy after abnormal FIT. Multivariate analysis demonstrated that females (odds ratio [OR] 1.26, confidence interval [CI]_95_, 1.07–1.47, *P* value = 0.0062) and those who spoke Spanish (OR 1.28, CI_95_ 1.3–2.3, *P* value < 0.0001) or another language other than English (OR 1.72, CI_95_ 1.3–2.3, *P* value < 0.0001) were more likely to undergo a recommended follow-up colonoscopy.

### Colonoscopy adherence

Of all the completed colonoscopies in our sample, 1,093 (82%) were within 6 months and 247 (18%) were after the 6-month recommended timeframe. The frequency of colonoscopy completion for abnormal FIT within 6 months increased significantly from 40.6% (n = 493) in 2017 without navigation to 46% (n = 600) in 2018 with navigation (*P* < 0.0001). Ninety-one percent of those patients who completed their colonoscopies in 2018 with PN were able to do so within the recommended 6-month period, compared with only 72% in 2017 without PN (*P* < 0.0001). With the help of the PN program, patients with a positive FIT test were 4 times more likely to complete a recommended colonoscopy within 182 days (OR 3.98, CI_95_, 2.86–5.39; *P* value < 0.0001). After adjusting for patient factors (age, gender, ethnicity, language, and distance), the significance of the PN intervention persists (OR 3.81, CI_95_, 2.8–5.2, *P* value < 0.0001) (Table 1).

**Table 1.**
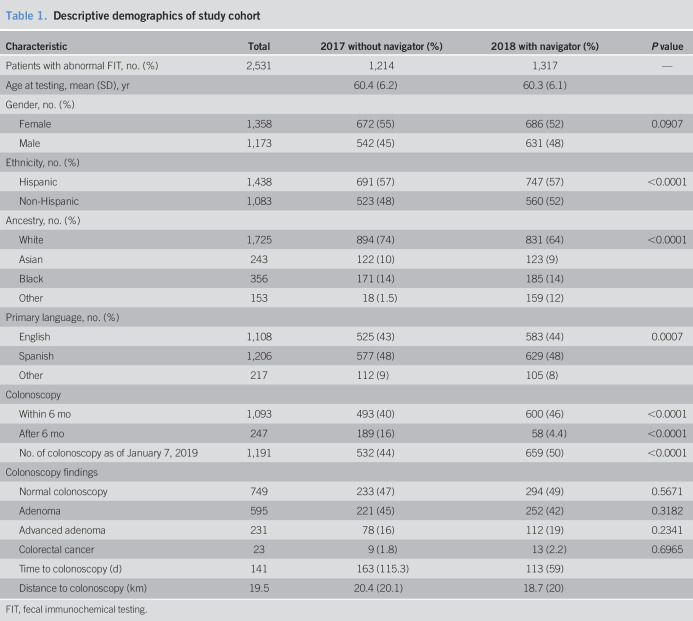
Descriptive demographics of study cohort

FIT, fecal immunochemical testing.

### Time to colonoscopy

**Figure 2.**
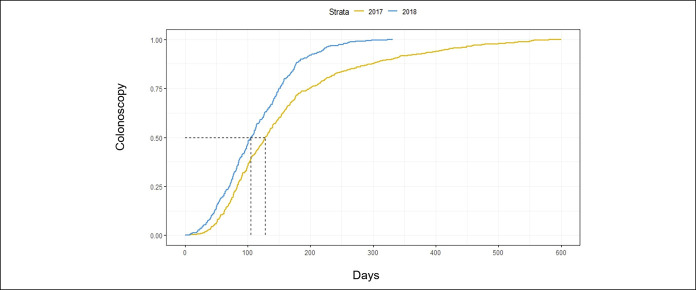
Proportional hazards effect of navigation on time (ds) from abnormal fecal immunochemical testing to colonoscopy.

Overall, the median time from abnormal FIT to colonoscopy declined from 121 days in 2017 without PN (mean = 163 days, SD 115) to 105 days in 2018 with PN (mean = 113 days, SD 59), *P* value < 0.0001. This demonstrates a 13% decrease in median time to colonoscopy and 31% decrease in mean time. The shortening of 16 days in median time was significant in a COX proportional hazards model (hazard ratio [HR] 0.700, CI_95_ 0.620–0.788, *P* value < 0.0001), showing that navigation decreased the overall length of time to colonoscopy in our sample and thereby decreased the hazard of completing a colonoscopy past the recommended 6-month period (Figure 2). This meaningful difference in time to colonoscopy persists even after adjusting for patient characteristics, including age, sex, race/ethnicity, language, and distance traveled (HR: 0.725, CI_95_ 0.642–0.881, *P* value < 0.0001).

### Distance to colonoscopy

**Figure 3.**
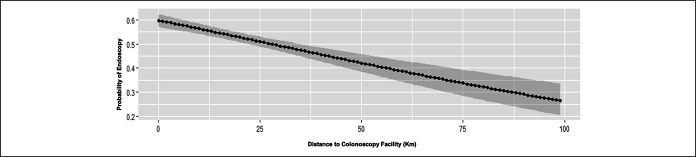
Probability of undergoing colonoscopy by distance (2017 and 2018).

Because a patient's distance to a colonoscopy treatment center could affect the likelihood of adhering to colonoscopy recommendation, we calculated the distance from each patient's home zip code to their LAC-DHS designated endoscopy facility (Figure 3). In 2017, the average distance traveled for colonoscopy per patient was 20.4 km (±20.1) as compared to 18.7 km (±20 km) in 2018. After adjusting for patient factors (age, sex, race/ethnicity, primary language), we found that for every km in distance, a patient was 1% less likely (OR 0.988, 95% CI, 0.984–0.992, *P* < 0.001) to come for their colonoscopy within 182 days.

### Colonoscopy findings

**Figure 4.**
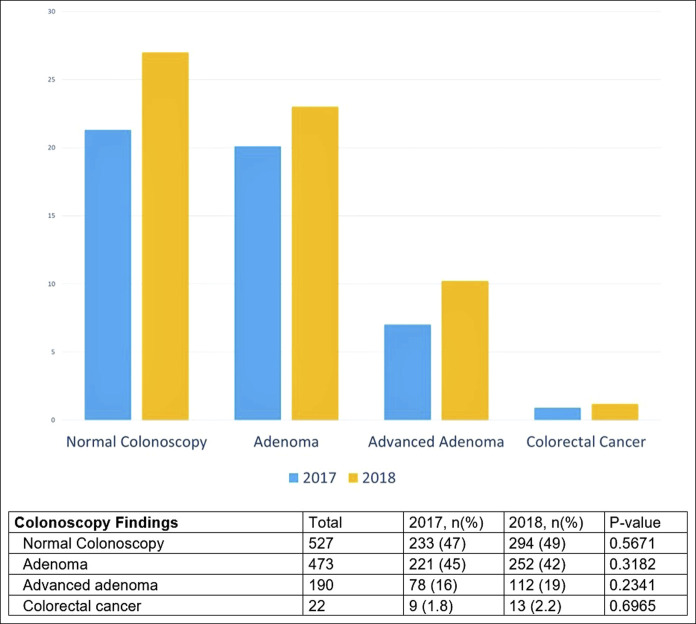
Frequency of colonoscopy findings in 2017 as compared to 2018.

Among those in 2018 who received PN and underwent colonoscopy (N = 600), more polyps were identified overall compared to 2017: 42% (n = 252) had adenomas, of which 20.2% (n = 112) had advanced polyps and 2.2% (n = 13) had CRC (Figure 4). Among those in 2017 who underwent colonoscopy but did not receive PN (N = 493), 45% (n = 221) had adenomas, 16% (n = 78) had advanced polyps, and 2% (n = 9) had CRC. After adjusting for patient factors (age, gender, ethnicity, language, and distance), multivariate analysis demonstrated that males tended to have more adenomas (OR 1.8, CI_95_ 1.4–2.3, *P* < 0.0001) and more advanced adenomas (OR 1.73, CI 1.2–2.4, *P* < 0.0001). For those with normal colonoscopy, we demonstrated after multivariate analysis that non–English-speaking, younger females tended to have colons with less advanced disease. We did not find a meaningful influence of PN on cancer detection likelihood in this study.

## DISCUSSION

In one of the largest and most ethnically diverse safety net healthcare systems in the nation, the implementation of a PN program led to a 6% increase in colonoscopy completion after abnormal FIT with a 16-day reduction in median time to colonoscopy. The PN program significantly impacted the timing of follow-up colonoscopy as patients were 4 times more likely to undergo the procedure within the recommended 6 months from abnormal FIT result in 2018 as compared to usual care in 2017. This improvement was seen among males and females and among all ethnic and racial groups.

Notably, we found that females and non-English speakers were more likely to undergo colonoscopy, which is consistent with previously reported results (18–20). Costas-Muniz et al. (21) reported in patient navigation study that participants who preferred Spanish were more likely to complete CRC screening (OR 1.64, CI 1.08–2.50) as compared to those who preferred English. Ellison et al. and Inadomi et al. similarly found that preference for speaking another language was associated with higher likelihood with CRC screening (19,22). By contrast, some studies found a positive correlation between higher acculturation to the US and higher CRC screening rates (16,23). Overall, the important conclusion from these findings is that PN may be more impactful for certain populations of patients (e.g. ethnicity, gender, and acculturation) and inform strategies to guide the utilization of resources.

In our subanalysis on the impact of distance from the patient's home to the colonoscopy facility, we found that longer travel distance was a barrier to completion of colonoscopy after adjusting for age, gender, and ethnicity. Qualitative studies on self-reported barriers to colon cancer screening in federally qualified health centers indicate driving long distances as a major logistical challenge that impede colonoscopy completion (24). High travel burdens have also been acknowledged by the Presidential Cancer Panel as a key barrier to healthcare access (25,26). There may be other logistical challenges including time commitment, availability of transportation, and availability of a chaperone that may contribute to the decrease in probability of adhering to colonoscopy (24,27). However, our findings are supported by a previous study of the National Cancer Database that demonstrated patients who travel longer distances to their designated healthcare facility were more likely to present with metastatic colon cancer than those traveling shorter distances (28). Because of the impact of transportation and distance in accessing CRC screening, programs are making efforts to mitigate these disparities by adjusting colonoscopy referral accounting for distance to endoscopy center and as well as working with patients in setting up transportation.

There is little evidence from well-designed studies that exist on the efficacy or effectiveness of PN programs in CRC screening within safety net health systems. In a single-center randomized control trial of patients within a safety net hospital setting, a PN program significantly improved colonoscopy completion within 6 months of study enrollment with patients in the navigation arm 1.5 times more likely to complete their colonoscopy (29). Navigation programs for breast screening have succeeded in reducing disparities within underserved populations. Multiple breast screening studies demonstrate that PN programs reduce delays in diagnosis and improve follow-up for patients in high-risk, urban, safety net populations (30–34). Patient navigators can not only facilitate improved healthcare access and quality for underserved populations through advocacy and care coordination but also they can address barriers that result in noncompliance with treatment recommendations.

A large consortium study demonstrated that interval time to colonoscopy after abnormal FIT differs widely when comparing integrated healthcare systems (Kaiser Northern California and Southern California, Parkland Health and Hospital System, and Group Health), where delays in receipt of colonoscopy reflected a combination of organizational-level and individual-level barriers (35). Moreover, data from integrated health systems demonstrate the importance of reducing delays to follow-up colonoscopy. In a study of 70,124 patients with abnormal FIT within the Kaiser Permanente system, colonoscopy delays of ≥ 12-months were associated with a 2.3-fold increased risk of any CRC and a 3.2-fold increased risk of advanced stage CRC (4). Although healthcare systems with the shortest time to and highest percentage of follow-up were those with the extensive organizational systems to facilitate follow-up, public safety net healthcare systems face greater personal- and system-level barriers to completing follow-up colonoscopy (36). Previous research suggests that lack of referrals, patient nonadherence, and physician decision not to follow-up have all contributed to delays or noncompletion of follow-up colonoscopy (36,37). Our own experience also indicates that there are additional opportunities within our current colonoscopy workflow (e.g., precolonoscopy class) that could improve adherence to colonoscopy. The importance of setting organizational and structural processes to follow-up an abnormal FIT cannot be understated because randomized controlled trials and observational studies of quality improvement initiatives conclude that these processes can improve outcomes. Data suggest that a multifaceted approach of automatic notification to gastroenterologists after abnormal fecal blood test results and reducing GI backlog is effective in increasing the timeliness and adherence to follow-up colonoscopy (38–40). Furthermore, PN has shown to enhance screening when integrated with other organizational processes to provide care to underserved and minority populations (41).

There are aspects to the study that merit attention, such as the fact that our data were collected from a large, ethnically diverse safety net healthcare system that includes 6 large medical centers in Los Angeles County and represents more than 2,500 patients with abnormal FIT within the analysis. In addition, this study is one of the first to demonstrate that distance traveled is a significant barrier to completing colonoscopy because patients are less likely to complete their procedure for every kilometer they must travel. Of course, one must keep in mind that distance traveled in Los Angeles has different implications than other cities and counties across the nation. Some of the limitations of this study include our analysis of a single healthcare system design in a geographically constrained setting, which may limit the generalizability of our intervention. In addition, our analysis is limited to a 6-month follow-up. Nonetheless, our findings highlight PN as a promising strategy to close the gap in follow-up colonoscopy for patients with an abnormal FIT.

## CONFLICTS OF INTEREST

**Guarantor of the article:** Gregory E. Idos, MD, MS.

**Specific author contributions:** G.I.: conceptualization, data curation, formal analysis, investigation, methodology, project administration, supervision, validation, visualization, writing—original draft, and writing—review and editing. J.D.B.: methodology, formal analysis, investigation, visualization, and writing. S.H., C.G., S.S., A.M., D.D.K.: data curation, investigation, and writing—review and editing. K.J., C.G., M.H., C.M.: data curation and writing—review and editing. J.M.-P.: formal analysis, investigation, visualization, and writing. P.G.: conceptualization, data curation, investigation, and writing—review and editing. S.D.: conceptualization, data curation, investigation, methodology, project administration, supervision, and writing—review and editing.

**Financial support:** NIH Grant Award KL2TR000131.

**Potential competing interests:** G.I. received research funding from Myriad Genetics and Laboratories. The remaining authors report no relevant conflicts of interests.

**Previous presentation:** This study was presented at the American College of Gastroenterology Annual Meeting; October 2019; San Antonio, Texas.

**Study Highlights**

**WHAT IS KNOWN**

✓ Follow-up of abnormal fecal immunochemical testing (FIT) results with completion colonoscopy is underused. Previous studies suggest only 30% to 70% of abnormal FIT results are followed by colonoscopy. The lack of follow-up colonoscopy is associated with poor clinical outcomes.

**WHAT IS NEW HERE**

✓ Few studies have shown that patient navigation improves colonoscopy completion in public health settings. Within the large safety net health system of Los Angeles County Department of Health Services, we demonstrate that integration of a patient navigator program resulted in patients undergoing follow-up colonoscopy after abnormal FIT results, and this follow-up occurred within a shorter time interval than before the program.

**TRANSLATIONAL IMPACT**

✓ Patient navigation is a model of care that reduces the gap from abnormal FIT to colonoscopy during colorectal cancer screening. The integration of this intervention could help address barriers to obtaining colorectal cancer care among underserved communities at medical institutions across the nation.
